# Inhibition of hexokinases holds potential as treatment strategy for rheumatoid arthritis

**DOI:** 10.1186/s13075-019-1865-3

**Published:** 2019-04-03

**Authors:** Guanhua Song, Qiqi Lu, Hua Fan, Xiumei Zhang, Luna Ge, Ruisong Tian, Shiguan Wang, Tingting Feng, Jihong Pan, Jingjing Feng, Yabo Xiao, Xin Yi, Ningxin Ren, Lin Wang

**Affiliations:** 1 Institute of Basic Medicine, Shandong Academy of Medical Sciences, Jinan, China; 2 School of Medicine and Life Sciences, University of Jinan-Shandong Academy of Medical Sciences, Jinan, China; 3 Graduate Education Centre of Shandong Academy of Medical Sciences, Jinan, China; 4 Research Center for Medicinal Biotechnology, Key Laboratory for Rare and Uncommon Diseases of Shandong Province, Shandong Academy of Medical Sciences, #18877, Jingshi Road, Jinan, 250062 China; 5 Department of Pathology, Shandong University Medical School, Jinan, China; 6 School of Basic Medical Sciences, Shandong University, Jinan, China

**Keywords:** Rheumatoid arthritis, Inflammation, Hypoxia, Glycolysis, Hexokinase, Lonidamine

## Abstract

**Introduction:**

Abnormal glycolytic metabolism contributes to joint inflammation and destruction in rheumatoid arthritis (RA). We examine the expression and function of hexokinases in RA and evaluate the potential of their specific inhibitor for clinical treatment.

**Methods:**

Detection of HKs was assessed in synovial tissue by immunohistology and Western blot. SiRNA and a specific hexokinases inhibitor, lonidamine (LND), were used to evaluate the role of hexokinase-I/II (HK-I/II). Pro-inflammatory and glycolysis factors, cell viability, and apoptosis were assessed by ELISA, RT-qPCR, MTS, and flow cytometry. The clinical effects of LND on type II collagen-induced arthritis (CIA) in DBA-/1 mouse model was evaluated by scoring their clinical responses, synovitis, and cartilage destructions, and ELISA was employed to analyze the concentrations of antibody in the serum of CIA model.

**Results:**

HK-I/II expression and their activities increased in the synovium of RA compared with osteoarthritis (OA). Silencing HK-I/II (siHK-I/II) or LND treatment decreased the production of pro-inflammatory factors, such as IL-6, IL-8, CXCL9, CXCL10, and CXCL11, and cell viability, but induced cell apoptosis of RASFs. The expression of TNF-α and IL-1β of macrophage in response to LPS stimulation were depressed as well after treatment with siHK-I/II or LND. Furthermore, leucocyte infiltration co-cultured with RASFs was also suppressed after inhibiting the expression or activity of HK-I/II. These anti-inflammatory effects overlapped with their anti-glycolytic activities. Treatment with LND in mice with CIA decreased the production of antibodies against IgG1, IgG2a, and IgG2b and consequently attenuated joint inflammation and destruction.

**Conclusions:**

HK-I/II contribute to shape the inflammatory phenotype of RASFs and macrophages. LND may be a potential drug in treating patients with RA.

**Electronic supplementary material:**

The online version of this article (10.1186/s13075-019-1865-3) contains supplementary material, which is available to authorized users.

## Introduction

Rheumatoid arthritis (RA) is an autoimmune disease characterized by synovial proliferation and leukocyte extravasation leading to joint destruction [1]. The increased proliferation and rapid activation of cells in the inflamed joint require the switch of glucose metabolism to a highly metabolically glycolytic state, in order to maintain energy homoeostasis [2, 3]. Recently, an abnormal glucose metabolism was observed in the synovial fluid and synovium of RA, which is evidenced by increased glycolytic enzyme activity and its product, lactate, but diminished glucose [4]. Further study confirmed that glucose metabolism is altered in rheumatoid arthritis synovial fibroblasts (RASFs) and the switch to glycolysis apparently supports its highly proliferation, invasion, and consequent pannus formation [5]. In addition, macrophages, another important contributor to RA, also undergo a metabolic shift from oxidative phosphorylation to glycolysis, thereby demonstrating a proinflammatory phenotype [6]. In particular, glycolysis inhibition by 3-bromopyruvate (3-BrPa) or PFK15 administration could suppress the inflammatory behaviors of RASFs and also prevent severe inflammation, joint swelling, and cartilage damage in vivo [5, 7]. Therefore, blocking glycolysis might be beneficial for RA and the exploration of novel hub linking glycolytic metabolism and inflammation might be promising in RA treatment.

Glycolytic metabolism is modulated by membrane glucose transporters (GLUTs), hexokinases (HKs) activity, and fructose 2,6-bisphosphate levels [8–10]. HKs catalyze the first committed step of glucose metabolism and glucose transported through GLUTs on the plasma membrane is phosphorylated by HKs to produce glucose-6-phosphate (G-6P) [9]. There are four isoforms of HK-I, HK-II, HK-III, and HK-IV in mammalian tissues, and their main distributions differ in various tissues [11]. Among them, HK-I, HK-II, and HK-III have higher affinity for glucose compared to HK-IV (also known as glucokinase) [12]. Importantly, HK-I and HK-II were found to be overexpressed in many types of cancer [13–15], and specific inhibition of HK activity exhibits promising anti-cancer activities [16, 17]. Recently, HK-II was demonstrated to be related with the invasive and migratory phenotype of RASFs and holds potential as a treatment target for RA [18]. Here, we further identified that HK-I and HK-II were mainly expressed in the synovial tissues of RA patients when compared with those of OA patients, further supporting their likely involvement in RA.

In this study, we examine whether HKs mediate the pathological mechanism of RA. The obvious induction of HKs, especially HK-I and HK-II, in RASFs and macrophages in response to pro-inflammatory and hypoxic stimulation, and their stronger localized expression at inflammatory sites of synovial tissues from RA patients suggest that HK-I and HK-II are directly involved in the pathogenesis of RA. Furthermore, silencing HK-I and HK-II, especially HK-II, could disrupt the proliferative and inflammatory phases of RA. Importantly, blocking HKs with its inhibitor, LND, attenuated the onset and severity of arthritis in CIA model, indicating its potential toward pre-clinical application.

## Materials and methods

### Sample collection and cell preparation

Synovial tissues were collected during knee joint replacement surgery from patients with RA (*n* = 16, 10 female, age 39 to 70 years old, mean 53 years) and OA (*n* = 12, 4 female, age 37 to 75 years old, mean 59 years). All patients fulfilled the criteria for the classification of RA and OA, respectively [19, 20]. The RA patients exhibited a disease duration of 3 to 9 years. All patients gave fully informed written consent approved by the institutional ethics committee, and research was performed in accordance with the Declaration of Helsinki. Biopsies were either embedded for immunohistochemical analysis, established as ex vivo RA whole-tissue synovial explant cultures, or snap-frozen in liquid nitrogen for protein analysis. The Ethics Committee of Shandong Research Center for Medicinal Biotechnology approved this study (approval no. 2016-2019). RASFs were isolated from synovial biopsy specimens of patients with RA as previously described [21], and cells between passages 4 and 7 were used for further study.

### Transfection

Small interfering RNA (siRNA) for HK-I, HK-II, and negative control was sourced from Ruibo (Guangzhou, China). The procedures of siRNA transfection were performed as described previously [21]. Whole cell lysates were extracted to assess knockdown efficiency of HK-I and HK-II in RASFs by western blot, and the two siRNAs showing the most silencing efficiency (siHKI#1: GCACAACAATGCCGTGGTT; siHK-I#2: GTCGACGGATCTCTTTACA; siHKII#1: ACGACAGCATCATTGTTAA; siHK-II#2: CTGGCTAACTTCATGGATA) were used for further experiments. Non-specific negative control siRNAs (NC) were also designed and synthesized. Equal amount of siHKI#1 and siHKI#2 or siHKII#1 and siHKII#2 was mixed to avoid off-targeting effects. The Mock group was defined as that supplemented with the transfection reagent only.

### Cell treatment

RASFs and THP-1 were cultured overnight in medium containing 1% fetal cells and subsequently stimulated with IL-1β and TNF-α (R&D Systems), or cobalt chloride (CoCl_2_) or lipopolysaccharides (LPS, Sigma, St. Louis, MO, USA). The HK-II inhibitor, lonidamine (Selleckchem), dissolved in dimethyl sulphoxide at a concentration of 1 mM, was brought to the final concentration with complete medium. Information about the specificity of the HKs has been previously published [22].

### MTT assay and apoptosis analysis

MTT assay were performed as previously described [21]. To evaluate the effects of HKs on the apoptosis of RASFs, RASFs were transfected with siHK-I/II or siCtrl, LND (100 μM), or its vehicle control (dimethyl sulphoxide) for 24 h, respectively. Next, the cells were trypsinized and collected for detection of apoptosis with an Annexin V-FITC apoptosis detection kit according to the manufacturer’s protocol. The tissue samples were incubated with goat pre-immune serum (Maixin-Bio) or treated with the modification buffer without the addition of the antibody to determine antibody specificity.

### Immunohistochemistry (IHC) and confocal laser scanning microscopy

Synovial tissues from RA and OA patients were embedded in paraffin and sectioned for IHC examination using antibodies against HK-I, HK-II, HK-III, or HK IV, which were purchased from Proteintech. IHC was performed as described previously [23]. For immunofluorescence, cells were sequentially probed with primary antibodies and fluorescence-labeled secondary antibodies (Jackson ImmunoResearch) as described before [24]. Mitotracker Red CMXRos (Yeason, China) was applied for mitochondrion staining. Images were captured under a confocal microscope (FV3000, Olympus).

### Western blot

FLS or THP-1 was collected for protein extraction in an ice-cold lysis buffer. Western blotting was performed as described [24] with antiHK-I, antiHK-II, antiHK-III, or antiHK-IV (Proteintech, China). GAPDH and β-tubulin were from Santa Cruz Biotechnology (Santa Cruz, CA, USA) and used as the loading control.

### Real-time quantitative PCR

Total RNA isolated with TRIzol was reverse transcribed using the First-Strand cDNA Synthesis Kit (Invitrogen). Real-time quantitative PCR (RT-qPCR) was performed as previously described [23]. Relative quantification of gene expression was analyzed using Lightcycler-480 PCR technology (Roche Diagnostics, Lewes, UK) with preoptimized conditions. The primers for each gene were listed in Additional file 1: Table S1. β-Actin was used as the internal loading control. Relative mRNA levels were measured using the 2-Δ cycle threshold (2-ΔCT) method. Three independent experiments were completed, and each reaction was performed in triplicate. The specific product of the amplification was further confirmed by melting analysis.

### Enzyme-linked immunosorbent assay (ELISA)

RASFs and THP-1 were treated with pro-inflammatory factors at different concentrations following transfection with siHKs or siCtrl, LND, or its vehicle control. Then, the serum-free conditioned media was collected and centrifuged at 6000 rpm for 10 min to remove particulates at 4 °C. TNF-α, IL-1β, IL-6, and IL-8 levels in culture supernatants were measured using ELISA kits provided by R&D Systems (Minneapolis, MD, USA).

### Leucocyte migration assays

Leucocytes were isolated from fresh heparinized venous blood obtained from healthy volunteers. RASFs in the well were transfected with siRNA targeting HK-I and HK-II or its negative control, LND, or its vehicle overnight, respectively. Then, cells were stimulated with TNF-α or IL-1β for another 24 h. Leucocytes were allowed to migrate to the RASF supernatant for 6 h. Leucocytes were then recovered from the lower chamber by gentle pipetting and counted manually using a hemocytometer. Each condition was performed in triplicate.

### Hexokinase activity assay

Cells were lysed with a lysis buffer containing 15 mM Tris pH 7.8, 0.25 mM sucrose, 0.5 mM dithiothreitol (DTT), 1 mM aminohexanoic acid, 1 mM phenylmethylsulfonyl fluoride (PMSF), and 2 μg/mL leupeptin. The lysates were then sonicated (five times, 30 s each) in a water bath, followed by centrifugation at 2000*g* at 4 °C for 5 min. The cell extracts (50 μL) were added to 950 μL of reaction buffer (100 mM Tris-HCl, pH 7.8, 5 mM ATP, 10 mM MgCl_2_, 10 mM glucose, 0.4 mM NADP, and 0.15 U/mL of glucose-6-phosphate dehydrogenase (G6PD, Sigma-Aldrich)) and incubated at 37 °C. The HK enzymatic activity was monitored by measuring G6PD-dependent conversion of NADP to NADPH spectrophotometrically at 340 nm. The activity was presented as nanomoles of NADPH generated from 1 mg of protein per minute at 37 °C.

### Glucose consumption and lactate production

Glucose consumption and lactate levels were measured using the Lactate Assay Kit (Biovision, Milpitas, CA, USA) per the manufacturer’s instructions. RASFs were plated and treated with siHKs/siCtrl or lonidamine/vehicle for various time points. Then, culture medium was collected, and cells were counted. Glucose and l-lactate concentrations in the incubation media were periodically monitored. The concentrations were normalized on cell numbers.

### 2-NBDG [2-N-(7-nitrobenz-2-oxa-1,3-diazol-4-yl)amino)-2-deoxyglucose] glucose uptake

Cellular glucose uptake was quantified by the 2-NBDG assay using a microplate reader. Cells were plated in 24-well plates at a rate of 2 × 10^5^ cells per well, and after the treatments, 2-NBDG was added at 10 μM final concentration and incubated for 1 h at 37 C. Then, cells were washed twice with PBS, serum-free medium was added, and the fluorescence intensity was immediately measured in a microplate reader at an excitation wavelength of 485 nm and an emission wavelength of 530 nm. After being taken by the cells, 2-NBDG was converted to a non-fluorescent derivative (2-NBDG metabolite). A fair estimation of the overall glucose uptake was obtained by quantifying the fluorescence. The assay has been described elsewhere [25].

### Measurement of anti-collagen antibodies

Serum samples were collected on days 7, 14, and 21 post immunization, and the titers of anti-CII IgG Abs were measured by ELISA. Bovine CII (1 μg/mL, Chondrex, Inc., Redmond, WA, USA) was coated onto microtiter plates (Maxisorp; Nunc, Roskilde, Denmark) overnight at 4 °C. After blocking with 1% BSA in PBS, serially diluted serum samples were added and incubated for 1 h at room temperature. After washing, HRP-conjugated rabbit anti-mouse IgG1, IgG2a, or IgG2b Ab (Zymed Laboratories, San Francisco, CA) was added and incubated for 2 h at 37 °C. After washing, Ab binding was visualized using *o*-phenylenediamine (Sigma-Aldrich). A standard serum composed of a mixture of sera from arthritic mice was added to each plate in serial dilutions, and a standard curve was constructed. The standard serum was defined as 1 U, and the Ab titers of serum samples were determined by the standard curve.

### Induction and assessment of CIA

Bovine collagen type II (CCII) (2 mg/mL) was mixed with complete Freund’s adjuvant (CFA) (2 mg/mL of *Mycobacterium tuberculosis*; Chondrex, Inc.) and injected intradermally on day 0 at the base of the tail with 100 μL of emulsion into 8- to 11-week-old DBA/1J mice. On day 21, mice received an intraperitoneal booster injection with 100 μg of CCII in incomplete Freund’s adjuvant (IFA). To investigate the treatment efficacy of lonidamine at disease onset, at day 21 post immunization, mice were treated with 50 mg/kg of vehicle or LND for 10 days. The thickness of the hind paws was measured using vernier calipers. The clinical arthritis score was calculated as described previously [23]. Each paw score was based on the degree of swelling and periarticular erythema using a scale of 0–3 as follows: 0, no evidence of erythema or swelling; 1, erythema confined to one joint region only; 2, erythema and swelling limited to one joint region only; and 3, severe erythema and swelling extending from the ankle to the midfoot (tarsal) joint involving both joint regions. Scores from all four paws were added to provide a total score for each mouse. The maximum possible score per mouse was 12. Experiments were performed using 10 mice per group. In the histological analysis, hind paws were fixed in 10% buffered formalin for 48 h and decalcified in 15% EDTA. The paws were then embedded in paraffin, and 5-μm sagittal serial sections of whole hind paws were cut. Tissue sections were stained with hematoxylin and eosin. The mice were housed at an Institutional Animal Care and Use Committee (IACUC), and the Ethics Committee of Research Center for Medicinal Biotechnology of Shandong approved the study.

### Statistical analysis

SPSS15 system for Windows was used for statistical analysis. The arthritis scores and hind paw thickness was analyzed statistically with the Mann–Whitney *U* test. Other differences between experimental groups were analyzed by two-tailed Student’s *t* test.

Data are reported as means ± SDs. *p* values < 0.05 were considered statistically significant.

## Result

### Expression of HKs in synovium of RA patients

**Fig. 1:**
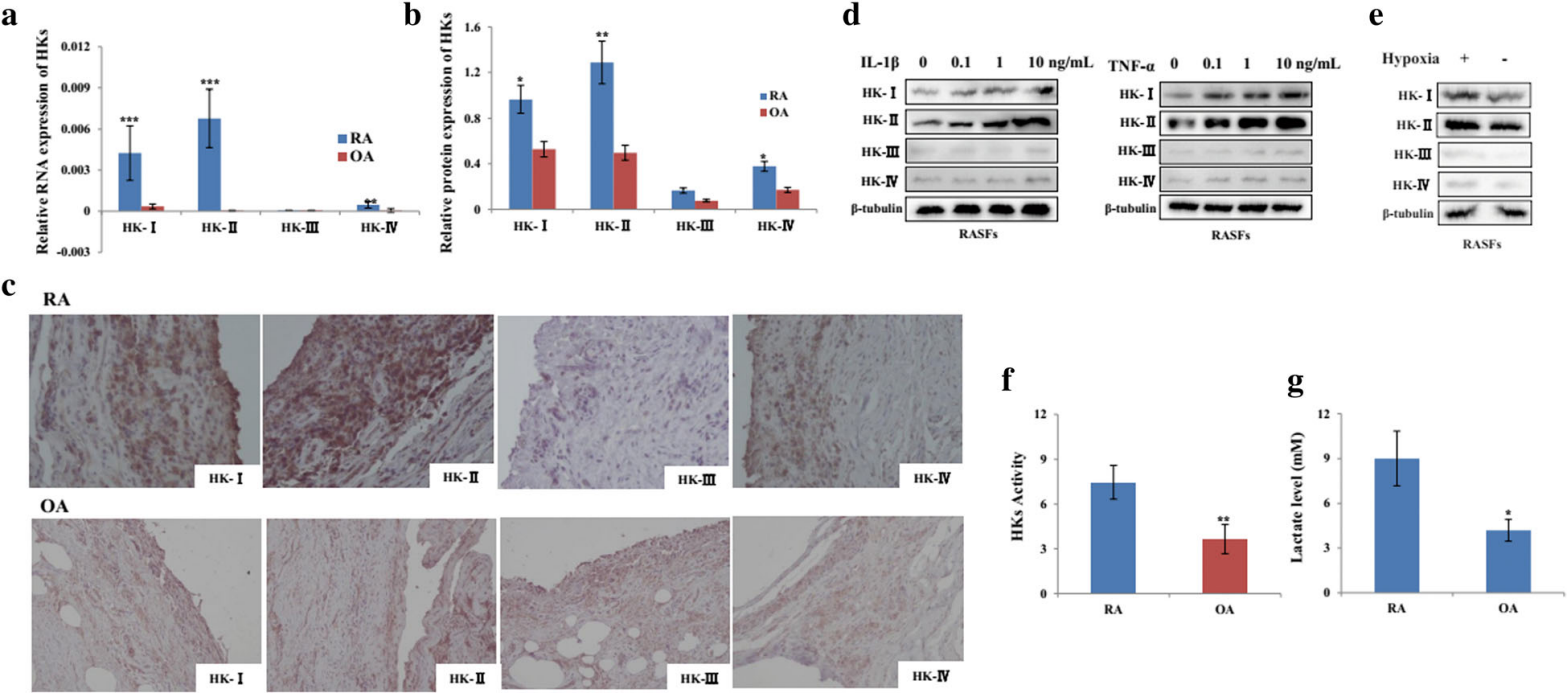
The expression and activity of HKs in synovial tissue from RA patients. **a**, **b** HKs mRNA and protein were detected in the synovial tissues from RA (*n* = 16) and OA (*n* = 12) patients by real-time PCR and Western blot. The relative expression ratio of HK protein in both groups is shown in the right. **c** Immunohistochemical staining of RA and OA synovial section using antibodies against HK-I, HK-II, HK-III, and HK-IV. Original magnification × 200. After stimulation with IL-1β (**d**, left), TNF-α (**d**, right), and CoCl_2_ (**e**, 10 μM)-induced hypoxia for 24 h, total protein was extracted from RASFs (*n* = 3) and subjected to Western Blot analysis for HK protein. **f** Hexokinase activity was determined in SFs from RA (*n* = 6) and OA (*n* = 6) patients and presented as nanomoles/milligram protein/min. **g** The lactate levels in the supernatant of RASFs (*n* = 6) and OASFs (*n* = 6) were determined by ELISA. Data represent as the mean ± SE. The statistical significance of differences between RA and OA group was determined. *p < 0.05, **p < 0.01, ***p < 0.001significantly different

To explore the role of HKs in RA, their expression in the synovial tissues derived from RA and OA patients were examined. The results showed that HKs expression were more abundantly expressed at mRNA (Fig. 1a) and protein (Fig. 1b) levels in RA patients compared with OA. Immunohistochemical analysis showed that HK-I, HK-II, and HK-IV expression was more localized in the synovial tissues from RA patients when compared with those from OA patients (Fig. 1c). As the implicated joints of RA are characterized by inflammatory and hypoxic condition, we subsequently analyzed the expression profiles of HKs in RASFs with the stimulation of pro-inflammatory factors and hypoxia. The data showed that, in response to IL-1β and TNF-α stimulation, the expression of HK-I and HK-II in RASFs increased steadily in a dose-dependent way at both RNAs (Additional file 2: Figure S1A and B) and protein (Fig. 1d) levels. Furthermore, hypoxic treatment could induce the expression of HK-I and HK-II at both RNAs (Additional file 2: Figure S1C) and protein levels as well (Fig. 1e). In contrast, no significant changes were observed in HK-III and HK-IV in response to the above treatment. We also measured the cytosol concentration of glucose-6-phosphate (G6P) and lactate release in culture medium of RASFs and OASFs. As shown in Fig. 1f and g, the basal levels of G6P and lactate were significantly higher in the culture medium from RASFs compared with OASFs. All these data suggest that HKs may be related with RA involving RASFs.

### HK-I and HK-II are crucial mediators for glycolysis and survival for RASFs

**Fig. 2:**
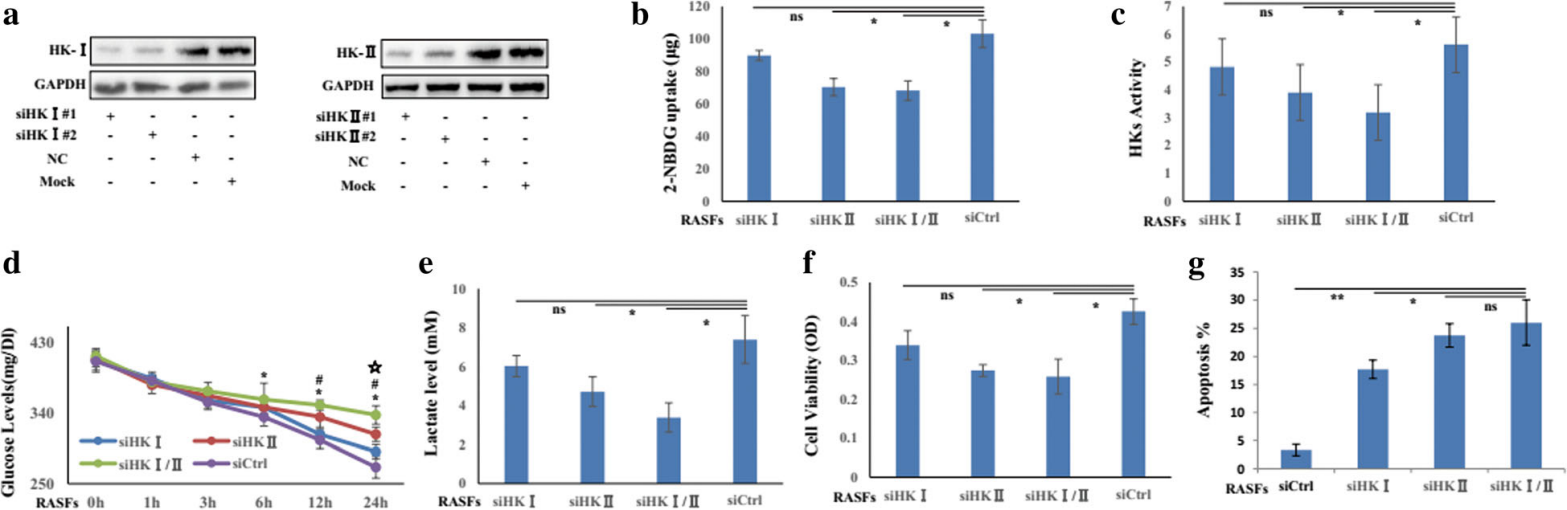
The effects of HKI/II on the glycolytic activities and cell survival of RASFs. **a** The silencing efficiency was confirmed by Western blot in RASFs (*n* = 3). After transfection with siHKI, siHKII or siHKI/II (50 nM) or its control (siCtrl) for 24 h, glucose uptake (**b**) and HKs activity (**c**) were determined in RASFs (*n* = 3). **d** Glucose concentrations in the incubation media of RASFs (*n* = 6) were periodically monitored. **e** l-lactate level was measured in the supernatant of RASFs (*n* = 6) after indicated treatments. The viability (**f**) and apoptosis (**g**) of RASFs (*n* = 3) were analyzed by MTS, Annexin X staining, and trypan blue. Data represent the mean ± SE. The statistical significance of differences between siCtrl and siHKI/II group was determined. *p < 0.05, **p < 0.01 significantly different; *ns* insignificant difference. Asterisk in **d** indicates siHKI/II vs siCtrl, number sign indicates siHKI vs siCtrl, and star indicates siHK II vs siCtrl (*p < 0.05, ^#^*p* < 0.05, and ^☆^*p* < 0.05)

As HK I and HK II are two main factors overexpressing in RASFs, we next examined the effects of HK-I and HK-II on glycolysis after silencing HK-I (siHK-I), HK-II (siHK-II), or both of them (siHK-I/II). The silencing efficiency of siRNA targeting HK I or HK II was confirmed by Western blot (Fig. 2a). As shown in Fig. 2, knocking down of HK-I/II inhibits glycolytic flux (Fig. 2b) and HK activity (Fig. 2c) in RASFs more obviously relative to silencing HK-I or HK-II alone. Similarly, treatment with siHK-I/II elicited significant decrease in glucose consumption (less glucose remained in the culture media) and lactate secretion (Fig. 2d, e). Moreover, silencing HKI/II could reduce cell viability (Fig. 2f), but induce apoptosis (Fig. 2g) of RASFs, suggesting that HK-I/II is functionally important for sustaining active glycolysis and survival of RASFs.

### LND, a HK II inhibitor, induces cell apoptosis of RASFs

**Fig. 3:**
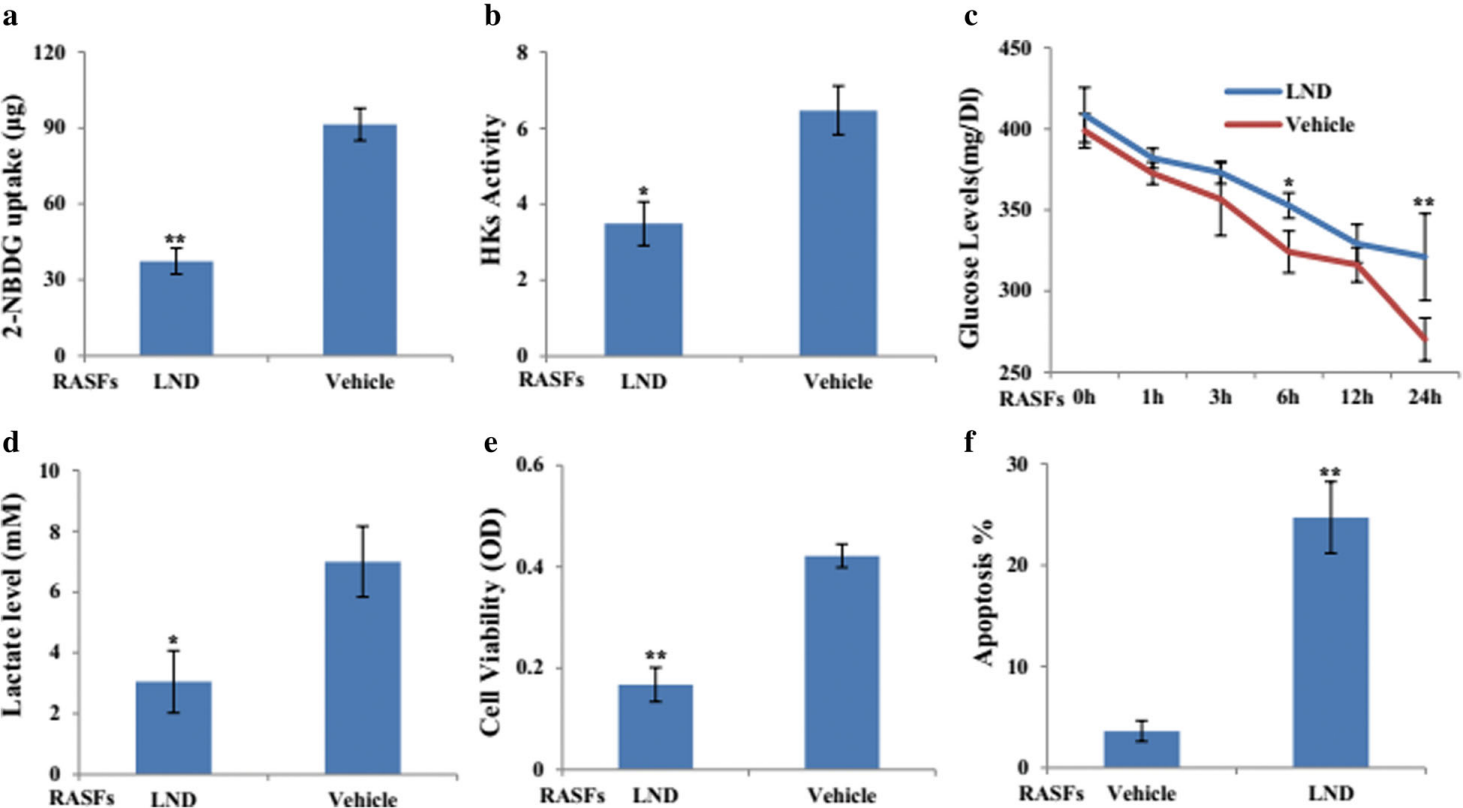
Treatment of HKs inhibitor, lonidamine, with RASFs. RASFs (*n* = 3) were treated with LND (100 μM) or its vehicle for 24 h, glucose uptake (**a**), HKs activity (**b**), glucose consumption (**c**), and l-lactate level (**d**) were determined. MTS and Annexin X staining were applied to detect the cell viability (**e**) and apoptosis (**f**) of RASFs (*n* = 3). The statistical significance of differences between LND- and vehicle-treated groups was determined. Data represent the mean ± SE. *p < 0.05, **p < 0.01 significantly different

Based on the above data, we determined the effects of HKII inhibitor, LND. Consistently, the glycolytic flux (Fig. 3a) and HK activity (Fig. 3b) of RASFs were downregulated in response to LND treatment. Moreover, glucose consumption (Fig. 3c) and lactate secretion (Fig. 3d) decreased significantly in LND-treated RASFs. Cellular localization of HK-I and HK-II in mitochondria was also suppressed in RASFs and THP-1 cells after LND treatment (Additional file 3: Figure S2). Additionally, LND treatment could inhibit cell viability (Fig. 3e), but induce cell apoptosis (Fig. 3f) of RASFs.

### Silencing HK-I/II inhibits the inflammatory activity of both RASFs and THP-1

**Fig. 4:**
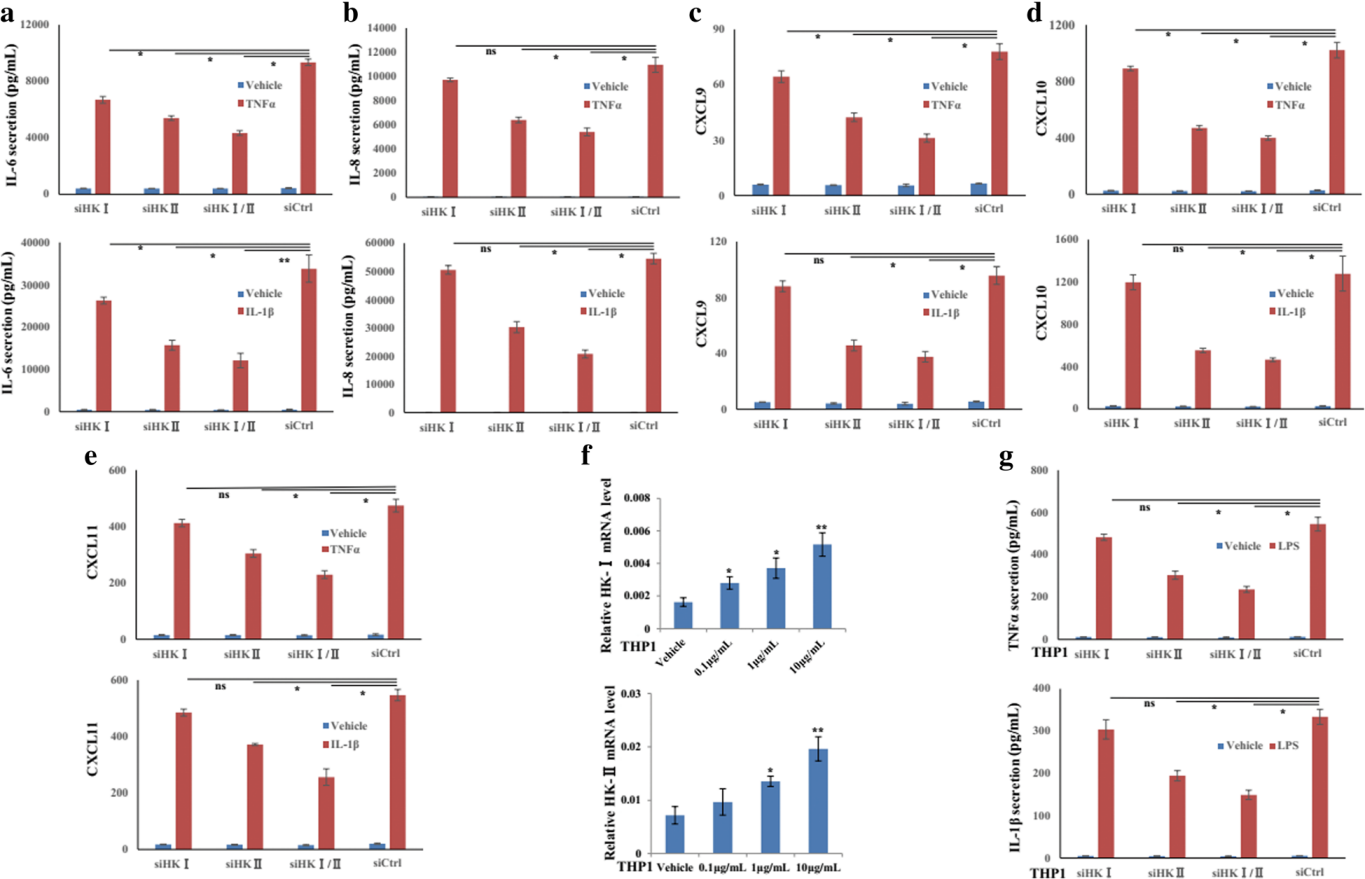
HKI/II regulate cytokine and chemokine production of RASFs and THP1. After transfection with siHKI, siHKII or siHKI/II or siCtrl (50 nM) for 6 h, RASFs (*n* = 3) were treated with IL-1β and TNF-α for 24 h. Secretion of IL-6 (**a**) and IL-8 (**b**) in culture supernatant was detected by ELISA. CXCL9 (**c**), CXCL10 (**d**), and CXCL11 (**e**) mRNA were assayed by RT-qPCR. **f** HK-I and II mRNA in THP1 cells were evaluated by RT-qPCR. **g** The production of TNF-α and IL-1β were also determined by ELISA in siHKI, siHKII or siHKI/II-transfected THP1 with the stimulation of LPS. Results in THP-1 are technical replicates. Data represent the mean ± SE. *p < 0.05, **p < 0.01 significantly different, *ns* insignificant difference

We next attempted to confirm the anti-inflammatory role of HK-I/II inhibition in RASFs. While there was no obviously decrease in the basal levels of IL-6 and IL-8, transfection with siHKI/II could attenuate IL-1β- or TNF-α-induced IL-6 (Fig. 4a) and IL-8 (Fig. 4b) production. Similar inhibition was also observed in siHK-I/II-transfected RASFs that attenuated the increase of CXCL9 (Fig. 4c), CXCL10 (Fig. 4d), and CXCL11 (Fig. 4e) induced by IL-1β or TNF-α.

Then, to determine whether HK-I and HK-II could affect the production of cytokines by macrophages, we treated THP-1 with LPS and the RT-qPCR analysis showed that HK-I and HK-II expression were strongly upregulated upon LPS treatment (Fig. 4f). To test the consequences of HK-I and HK-II induction in THP-1 cells, we measured TNF-α and IL-1β production in siHK-I, siHK-II, or siHK-I/II-transfected THP-1 cells with the stimulation of LPS for 24 h. Compared with the control, silencing HK-II or both HK-I and HK-II could significantly repress the release of IL-1β and TNF-α, in response to LPS (Fig. 4g). Silencing efficiency of siRNA targeting HK-I and II was provided in Additional file 4: Figure S3. These data indicated that inhibition of HK-I/II demonstrated an anti-inflammatory role in RA.

### LND suppresses the production of inflammatory factors in both RASFs and THP-1 cells

**Fig. 5:**
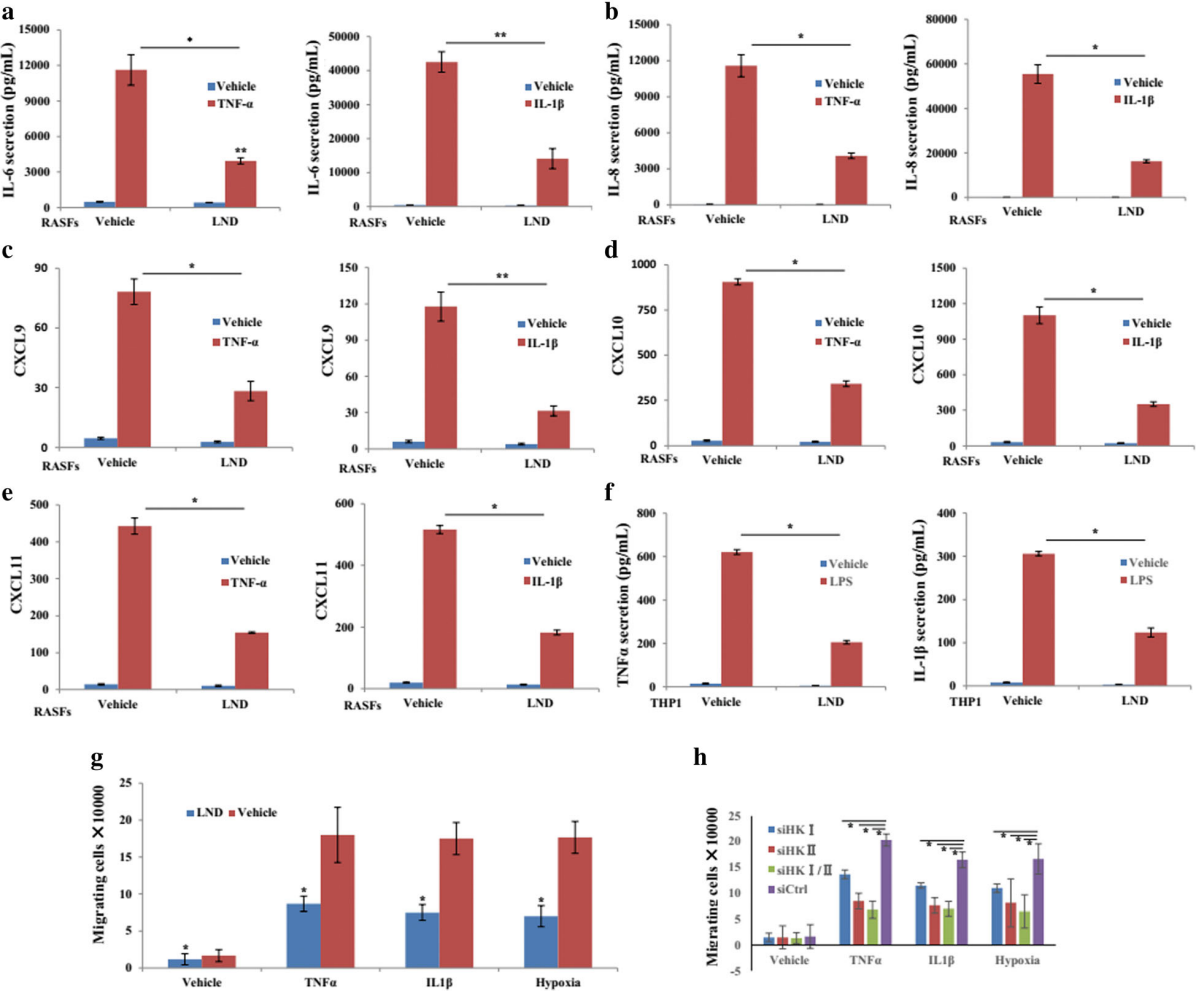
Effects of lonidamine on the inflammatory activity of RASFs and THP1. RASFs (*n* = 3) were incubated with LND (100 μM) overnight, followed by IL-1β (10 ng/mL) or TNF-α (10 ng/mL) for 24 h. Then, cell culture medium was collected and subjected to ELISA analysis for IL-6 (**a**) and IL-8 (**b**). CXCL9 (**c**), CXCL10 (**d**), and CXCL11 (**e**) mRNA were assayed by RT-qPCR. **f** After stimulation with LPS (1 μg/mL) for 6 h, THP-1 was treated with LND (100 μM) for another 24 h and the supernatant was collected for analyzing the release of TNF-α and IL-1β by ELISA. RASFs were incubated in presence or absence of LND/vehicle (**g**), or siHK-I/II/siCtrl (**h**), and stimulated with TNF-α, IL-1β, or CoCl_2_ for 24 h. Peripheral blood leucocytes from healthy donors were incubated with conditioned medium from RASFs in a transwell system for 6 h, and the number of migrating leucocytes was counted. Results in THP-1 are technical replicates, and data are shown as the mean ± SEM. *p < 0.05, **p < 0.01significantly different

Then, we evaluated the anti-inflammatory effect of LND. The data showed that LND treatment could suppress the basal, as well as TNF-α or IL-1β-induced IL-6 (Fig. 5a) and IL-8 (Fig. 5b) production. Similar effects on CXCL9 (Fig. 5c), CXCL10 (Fig. 5d), and CXCL11 (Fig. 5e) were also observed in RASFs with the treatment with LND, followed by TNF-α or IL-1β stimulation. We next analyzed the effect of LND on cytokine production of THP-1. LND could also inhibit the release of IL-1β and TNF-α with the stimulation of LPS (Fig. 5f). More importantly, LND treatment could reduce the basal levels of IL-1β and TNF-α.

### LND reduces the chemo-attractant abilities of RASFs

To examine potential effects of LND on the ability of RASFs to recruit immune cells, we treated RASFs overnight in the absence or presence of LND in medium alone or in combination with inflammatory stimuli. Cell-free supernatants were collected and used as a chemotactic source for healthy donor peripheral blood leucocytes. As expected, based on gene expression data, LND significantly reduced the chemo-attractant potential of RASFs in response to TNF-α, IL-1β, or hypoxia (Fig. 5g), and similar trends were observed in RASFs transfected with siHK-I/II (Fig. 5h). Together, these data indicate that changes in RASF gene expression following siHK-I/II or LND exposure are enough to modulate the chemo-attractant potential of RASFs.

### Treatment of CIA with LND

**Fig. 6:**
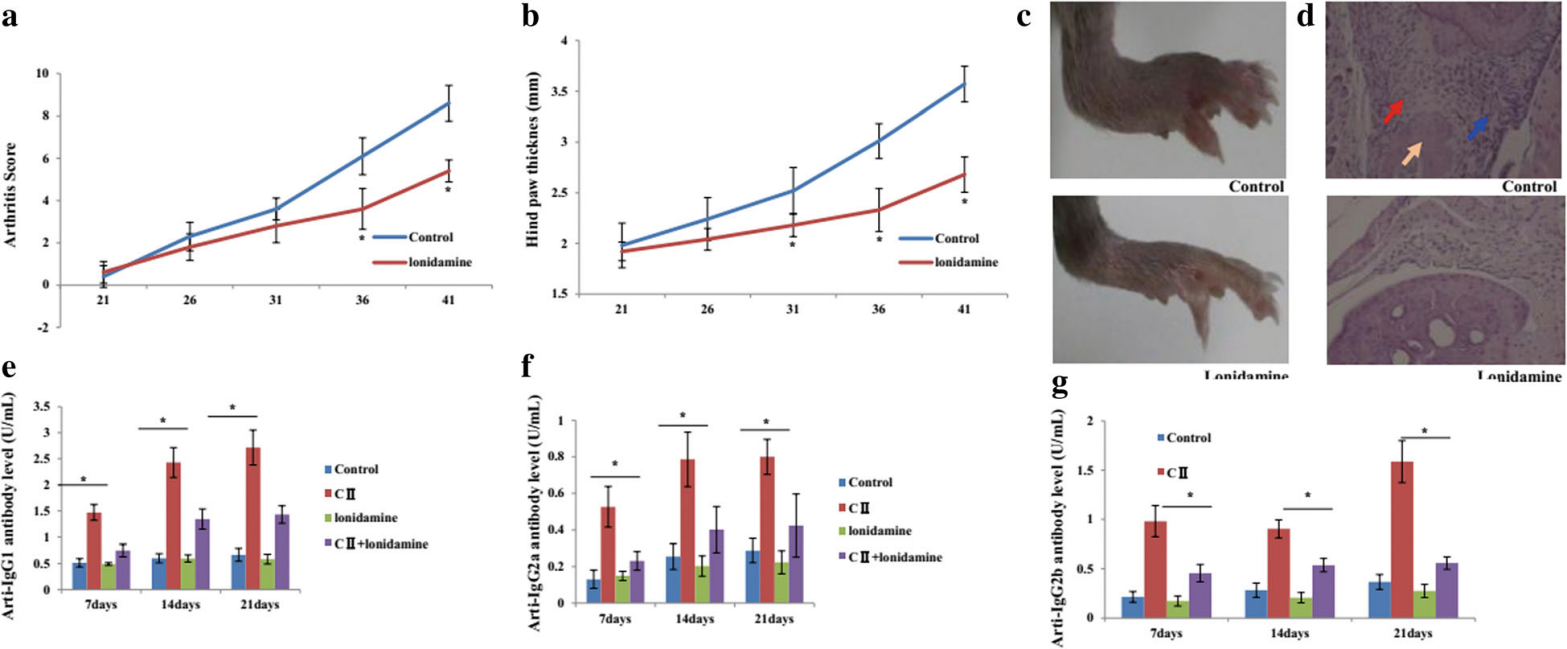
Antiarthritic effects of Lonidamine on collagen-induced arthritis. Clinical severity (**a**) and hind paw thickness (**b**) of CIA following treatment with LND or its vehicle. Clinical scores and hind paw thickness were monitored daily for 3 weeks after the booster injection (*n* = 10 per group). **c** Paw photographs obtained on day 42 from mice with CIA treated with vehicle or LND. The symptoms of CIA were observed in the hind paws of these collagen-treated mice as indicated treatments. **d** Photomicrographs show ankle joints were harvested on day 21 after the second immunization, sectioned, and stained with hematoxylin and eosin. Synovial hyperplasia (red arrow), immune cell infiltration (blue arrow), and bone destruction (yellow arrow) can be seen. Serum levels of anti-CII IgG1 (**e**), IgG2a (**f**), and IgG2b (**g**) in the CIA mice treated with vehicle or LND were measured by ELISA on days 7, 14, and 21 after the booster immunization. The statistical significances of differences between LND treated group and vehicle group were determined. Data represent the mean ± SE. *p < 0.05significantly different

To assess the antiarthritic effects of LND, we immunized DBA/1J mice with bovine CII and administered LND orally at doses of 100 mg/kg. When LND administration was started before the onset of the disease, 21 days after the initial immunization for 10 days, LND mitigated the clinical manifestation of CIA, such as arthritis score (Fig. 6a), hind paw thickness (Fig. 6b) and swelling (Fig. 6c). Histological examination of the joints from the vehicle-treated mice revealed synovial hyperplasia, mononuclear cell infiltration, and bone destruction were all ameliorated in the mice treated with LND (Fig. 6d).

To investigate the influence of LND treatment on the humoral immune response to collagen, sera obtained from mice at 7, 14, and 21 days after the second immunization. The serum levels of anti-CII IgG1 (Fig. 6e), IgG2a (Fig. 6f), and IgG2b (Fig. 6) were dramatically increased in response to the second immunization, but all of these responses were significantly suppressed by LND treatment.

## Discussion

Based on the recently published data that HK-II represents as a possible therapeutic target for RA [18], in this study, we aimed to examine the expression and roles of HKs in RA. We found that HK-I and HK-II are more overexpressed in the synovial tissues from RA relative to OA ones and localize to lining and sublining layer regions. Furthermore, HK-I and HK-II could be induced more obviously in RASFs by hypoxia and pro-inflammatory factors. Thus, HK-I and HK-II may be responsible for regulating RASFs activity. Our data further supports a therapeutic potential for targeting HKs in RA, showing that silencing HKs, especially HK-II, suppresses TNF-α-, IL-1β-, or TLR ligand-induced expression of key molecules in RASFs and macrophages that contribute to pathology, including cytokines and chemokine. These changes in expression levels translated directly into functional consequences for cellular behaviors of RASFs, including reduced proliferation and cytokine production, diminished capacity to recruit immune cells, and increased cell apoptosis and death. In attempts to validate the applicability of targeting HKs in RA, a specific HKs inhibitor, LND, was further utilized and we observed some similar, but even more obviously suppressive effects on the inflammatory phenotype of RA. We have also demonstrated that LND ameliorated the disease in an animal model of RA and it may constitute a new class of antirheumatic drugs that act additively with biological therapies targeting immunity. Investigation of HKs in the treatment of rheumatic diseases will be facilitated by the fact that such drugs have already been tested in patients with cancer.

RASFs not only constitute a cellular mesh for the inflammatory process that characterizes RA, but also acquire a permanently aggressive, tumor-like phenotype that can initiate and perpetuate RA [26]. Of note, tumor cells rely on a peculiar shift to aerobic glycolysis-dependent metabolism (the Warburg effect) as the main energy source to sustain their uncontrolled growth and proliferation [22]. Since Warburg metabolism is also relevant to inflammation, the approaches disrupting it may also be applicable for treating inflammatory disorders [27]. In RA, it is recently reported that the balance between glycolysis and oxidative phosphorylation was shifted toward glycolysis in RASFs [2–6]. Importantly, inhibition of the glycolytic activity of 6-phosphofructo-2-kinase/fructose-2, 6-bisphosphatase 3 (PFKFB3) not only impaired cytokine secretion and decreased cell proliferation and migration of RASFs, but also ameliorated the onset and severity in an arthritis model [7]. Therefore, blocking glycolysis in RASFs might be a feasible strategy for RA treatment. In this study, our data showed that specific silencing of HK-I/II could inhibit the production of cytokines and chemokine in response to pro-inflammatory factors, thus providing evidence that HK-I/II may be related with the inflammatory phenotype of RASFs.

Hypoxia is important in driving inflammatory responses of RA that could activate the genes encoding nearly every step of glycolysis [28]. In response to hypoxia, the residing cells in joints, such as RASFs and macrophages, produced a large amount of proinflammatory factors that foster monocyte recruitment, neovascularization and bone destruction [28, 29]. Especially, glycolysis and inflammation are closely related in macrophage and mainly depend on the effect of hypoxia on glucose uptake and HK-I/II activities [6]. In fact, it has been demonstrated that HK2 activation and glycolytic activity are required for protecting tumor cells, such as DLBCL, lung cancer, and prostate cancer, to enter into an adaptive process under hypoxic stress [30–32]. Hypoxia, glycolysis, and inflammation are hence interconnected during RA progression. In this study, HK-I/II expression in RASFs and THP1-derived macrophages could be induced by overlapping inflammatory factors and hypoxia, indicating that the glycolytic activity mediated by HK-I/II is required for sustaining the inflammatory phenotype under hypoxic condition in RA.

HK I and HK II differ in their affinities for glucose and ATP [11]. Besides facilitating coupling glycolysis and oxidative phosphorylation, HK-II indirectly regulates non-oxidative ribonucleotide synthesis and glutamine-dependent anapleurosis [12]. Furthermore, HK-I and HK-II protect against mitochondrial-regulated apoptosis through direct interaction with VDAC in mitochondria [33, 34]. HK-II localizing in mitochondria could also antagonize Bax/Bak-mediated apoptosis^34^ and inhibit Ca^2+^- and ROS-induced mitochondrial permeability transition pore (mPTP) opening and cell death [35, 36]. In addition, the dual regulation that mTORC1 stimulates HK-II expression while HK-II inhibits mTORC1 under starvation to stimulate autophagy, provides an adaptive mechanism to preserve cellular homeostasis dependent on metabolic status [37]. In fact, the dynamic change of HK-II expression directly impact cellular glucose metabolism in various diseases [11, 38] and HK-II constitutes an attractive potential selective target for RA as its regulation on invasive ability of SFs [18]. In this study, HKs, especially HK-II, were required for survival and proliferation for RASFs, further supporting the likelihood as treatment target in RA.

LND, a derivative of indazolcarboxilic acid, is a glycolytic inhibitor through directly inactivating HKs [38]. Its anti-cancer effect has been extensively studied by inhibiting glucose metabolism, suppressing succinate-induced respiration of mitochondria, inducing cellular reactive oxygen species (ROS), and thereby leading to intracellular acidification and decrease of NADPH, glutathione, and ATP generation [39, 40]. It could also targets mPTP to induce release of apoptotic factors [40, 41]. Tumor selectivity and low toxicity to normal tissues are critical characteristics that make LND an attractive agent for tumor treatment [41, 42]. In this study, our observation that LND exhibits the anti-inflammatory or anti-proliferation activities on RASFs and macrophages supports that specific targeting HKs may have therapeutic potential in blocking the activation of multiple cell types important in pathology in RA. Importantly, LND treatment in vivo could ameliorate the severity in CIA model, further supporting its feasibility for clinical application. More importantly, in tumor, LND could potentiate the activity of conventional chemotherapeutic agents and also enhance response to targeted therapeutics [40, 43]. As DMARDs are widely used in RA but patients receiving higher doses are at a higher risk of serious immunosuppression and infection [44], further research will elucidate whether LND exerts additive effects with DMARDs and the drug doses used in humans with RA. Furthermore, since the anti-CII Abs have been implicated in the pathogenesis of CIA [45], the reduced production of anti-CII Abs might also be responsible for the ameliorating effect of LND treatment. Further study would still be needed to clarify the effect of LND on acquired immune responses.

As the gender imbalance (62% females in RA group vs 33% in OA group) occurred in our sample collection, further study would still be needed in more clinical samples to exclude its possible contribution to the different expression profile of HKs in RA and OA patients. Furthermore, as females are more susceptible to RA, studies are also ongoing to detect the effects of gender on HKs expression and estrogen was chosen firstly.

## Conclusion

In summary, our study provides direct evidence of HK-I/II in the regulation of pro-inflammatory mechanisms in RA. The inhibitory effects of HK-I/II and its inhibitor on RASFs and macrophages could provide a dual means of affecting RA and further support a role for LND in the treatment of RA.

## Additional files

Additional file 1: **Table S1.** Primers used in this study. (DOC 31 kb)

Additional file 2: **Figure S1.** Effects of proinflammatory factors and hypoxia on the mRNA level of HKs. RASFs (*n* = 3) were treated with IL-1β (A), TNF-α (B) and CoCl_2_ (C). Then, RT-qPCR was performed to detect the mRNA expression of HK-I, HK-II, HK-III and HK-IV. Results are shown as the mean ± SEM. The statistical significances of differences vs vehicle or control group were determined. *p < 0.05, **p < 0.01 significantly different. (TIF 27758 kb)

Additional file 3: **Figure S2.** Effects of LND on cellular distribution of HK-I and HK-II. RASFs (A) and THP-1 (B) cells were treated with LND (100 μM) for 24 h. Immunofluorescent staining (green) of HK-I and HK-II in mitochondria (blue) was analyzed. (TIF 2967 kb)

Additional file 4: **Figure S3.** Silencing efficiency of siRNA targeting HK-I (A) and HK-II (B) in THP-1 cells. (TIF 849 kb)

## Abbreviations

3-BrPa: 3-Bromopyruvate
CIA: Collagen-induced arthritis
CoCl2: Cobalt chloride 2
DMEM: Dulbecco’s modified Eagle’s medium
DTT: Dithiothreitol
ELISA: Enzyme-linked immunosorbent assay
FBS: Fetal bovine serum
G-6P: Glucose-6-phosphate
GAPDH: Glyceraldehyde-3-phosphate dehydrogenase
HK: Hexokinase
IgG: Immunoglobulin G
IHC: Immunohistochemistry
IL-1β: Interleukin 1β
LND: Lonidamine
MTT: Methylthiazolyldiphenyl-tetrazolium bromide
OA: Osteoarthritis
PBS: Phosphate-buffered saline
PMSF: Phenylmethylsulfonyl fluoride
PVDF: Polyvinylidene difluoride
RA: Rheumatoid arthritis
RASFs: Synovial fibroblasts of rheumatoid arthritis
RT-qPCR: Real-time quantitative PCR
SDS-PAGE: Sodium dodecyl sulphate polyacrylamide gel electrophoresis
siRNA: Small interfering RNA
TNF-α: Tumor necrosis factor-α

## References

[1] Ganesan R, Rasool M (2017). Fibroblast-like synoviocytes dependent effector molecules as a critical mediator for rheumatoid arthritis: current status and future directions. Int Rev Immunol.

[2] Bustamante MF, Garcia-Carbonell R, Whisenant KD, Gu'ma M (2017). Fibroblast-like synoviocyte metabolism in the pathogenesis of rheumatoid arthritis. Arthritis Res Ther.

[3] Pucino V, Bombardieri M, Pitzalis C, Mauro C (2017). Lactate at the crossroads of metabolism, inflammation, and autoimmunity. Eur J Immunol.

[4] Chang X, Wei C (2011). Glycolysis and rheumatoid arthritis. Int J Rheum Dis.

[5] Garcia-Carbonell R, Divakaruni AS, Lodi A, Vicente-Suarez I, Vicente-Suarez I, Saha A, Cheroutre H (2016). Critical role of glucose metabolism in rheumatoid arthritis fibroblast-like synoviocytes. Arthritis Rheumatol.

[6] Kelly B, O'Neill LA (2015). Metabolic reprogramming in macrophages and dendritic cells in innate immunity. Cell Res.

[7] Zou Y, Zeng S, Huang M, Qiu Q, Xiao Y, Shi M (2017). Inhibition of 6-phosphofructo-2-kinase suppresses fibroblast-like synoviocytes mediated synovial inflammation and joint destruction in rheumatoid arthritis. Br J Pharmacol.

[8] Ancey PB, Contat C, Meylan E. Glucose transporters in cancer from tumor cells to the tumor microenvironment. FEBS J. 2018. https://www.ncbi.nlm.nih.gov/pubmed/29893496. 10.1111/febs.14577 29893496

[9] Robey RB, Hay N (2006). Mitochondrial hexokinases, novel mediators of the antiapoptotic effects of growth factors and Akt. Oncogene.

[10] Rider MH, Bertrand L, Vertommen D, Michels PA, Rousseau GG, Hue L (2004). 6-phosphofructo-2-kinase/fructose-2,6-bisphosphatase: head-to-head with a bifunctional enzyme that controls glycolysis. Biochem J.

[11] Wilson JE (2003). Isozymes of mammalian hexokinase: structure, subcellular localization and metabolic function. J Exp Biol.

[12] Roberts DJ, Miyamoto S (2015). Hexokinase II integrates energy metabolism and cellular protection: Akting on mitochondria and TORCing to autophagy. Cell Death Differ.

[13] Mathupala SP, Ko YH, Pedersen PL (2006). Hexokinase II cancer’s double-edged sword acting as both facilitator and gatekeeper of malignancy when bound to mitochondria. Oncogene.

[14] Marín-Hernández A, López-Ramírez SY, Del Mazo-Monsalvo I (2014). Modeling cancer glycolysis under hypoglycemia, and the role played by the differential expression of glycolytic isoforms. FEBS J.

[15] Marín-Hernández A, López-Ramírez SY, Del Mazo-Monsalvo I, Gallardo-Pérez JC, Rodríguez-Enríquez S, Moreno-Sánchez R (2009). Reciprocal immunohistochemical expression of sodium/iodide symporter and hexokinase I in primary thyroid tumors with synchronous cervical metastasis. Laryngoscope.

[16] Ganapathy-Kanniappan S, Vali M, Kunjithapatham R, Buijs M, Syed LH, Rao PP (2010). 3-Bromopyruvate: a new targeted antiglycolytic agent and a promise for cancer therapy. Curr Pharm Biotechnol.

[17] Kurtoglu M, Maher JC, Lampidis TJ (2007). Differential toxic mechanisms of 2-deoxy-D-glucose versus 2-fluorodeoxy-D-glucose in hypoxic and normoxic tumor cells. Antioxid Redox Signal.

[18] Bustamante MF, Oliveira PG, Garcia-Carbonell R, Croft AP, Smith JM, Serrano RL (2018). Hexokinase 2 as a novel selective metabolic target for rheumatoid arthritis. Ann Rheum Dis.

[19] Arnett FC, Edworthy SM, Bloch DA, Mcshane DJ, Fries JF, Cooper NS (1988). The American Rheumatism Association 1987 revised criteria for the classification of rheumatoid arthritis. Arthritis Rheum.

[20] Altman R, Asch E, Bloch D, Bole G, Borenstein D, Brandt K (1986). Development of criteria for the classification and reporting of osteoarthritis. Classification of osteoarthritis of the knee. Diagnostic and Therapeutic Criteria Committee of the American Rheumatism Association. Arthritis Rheum.

[21] Wang L, Song G, Zheng Y, Hang R, Pan J, Han J (2015). Expression of Semaphorin 4A and its potential role in rheumatoid arthritis. Arthritis Res Ther.

[22] Bose S, Le A (2018). Glucose metabolism in cancer. Adv Exp Med Biol.

[23] Wang L, Zheng Y, Xu H, Yan X, Chang X (2013). Investigate pathogenic mechanism of TXNDC5 in rheumatoid arthritis. PLoS One.

[24] Wang L, Song G, Zhang X, Feng T, Pan J, Chen W (2017). PADI2-mediated citrullination promotes prostate cancer progression. Cancer Res.

[25] Zou C, Wang Y, Shen Z (2005). 2-NBDG as a fluorescent indicator for direct glucose uptake measurement. J Biochem Biophys Method.

[26] Bottini N, Firestein GS (2013). Duality of fibroblast-like synoviocytes in RA: passive responders and imprinted aggressors. Nat Rev Rheumatol.

[27] Palsson-McDermott EM, O'Neill LA (2013). The Warburg effect then and now: from cancer to inflammatory diseases. Bioessays.

[28] Konisti S, Kiriakidis S, Paleolog EM (2012). Hypoxia--a key regulator of angiogenesis and inflammation in rheumatoid arthritis. Nat Rev Rheumatol.

[29] Quiñonez-Flores CM, González-Chávez SA, Pacheco-Tena C (2016). Hypoxia and its implications in rheumatoid arthritis. J Biomed Sci.

[30] Bhalla K, Jaber S, Nahid MN, Underwood K, Beheshti A, Landon A (2018). Role of hypoxia in diffuse large B-cell lymphoma: metabolic repression and selective translation of HK2 facilitates development of DLBCL. Sci Rep.

[31] Ma Y, Yu C, Mohamed EM, Shao H, Wang L, Sundaresan G (2016). A causal link from ALK to hexokinase II overexpression and hyperactive glycolysis in EML4-ALK-positive lung cancer. Oncogene.

[32] Wang L, Xiong H, Wu F, Shao H, Wang L, Sundaresan G (2014). Hexokinase 2-mediated Warburg effect is required for PTEN- and p53-deficiency-driven prostate cancer growth. Cell Rep.

[33] Abu-Hamad S, Zaid H, Israelson A, Nahon E, Shoshanbarmatz V (2008). Hexokinase-I protection against apoptotic cell death is mediated via interaction with the voltage-dependentanionchannel-1: mapping the site of binding. J Biol Chem.

[34] Majewski N, Nogueira V, Bhaskar P, Coy PE, Skeen JE, Gottlob K (2004). Hexokinase mitochondria interaction mediated by Akt is required to inhibit apoptosis in the presence or absence of Bax and Bak. Mol Cell.

[35] Wu R, Wyatt E, Chawla K, Tran M, Ghanefar M, Laakso M (2012). Hexokinase II knockdown results in exaggerated cardiac hypertrophy via increased ROS production. EMBO Mol Med.

[36] Glass-Marmor L, Penso J, Beitner R (1999). Ca2+−induced changes in energy metabolism and viability of melanoma cells. Br J Cancer.

[37] Bhaskar PT, Nogueira V, Patra KC, Jeon SM, Park Y, Robey RB (2009). mTORC1 hyperactivity inhibits serum deprivation-induced apoptosis via increased hexokinase II and GLUT1 expression, sustained Mcl-1 expression, and glycogen synthase kinase 3beta inhibition. Mol Cell Biol.

[38] Rempel A, Mathupala SP, Griffin CA, Hawkins AL, Pedersen PL (1996). Glucose catabolism in cancer cells: amplification of the gene encoding type II hexokinase. Cancer Res.

[39] Nancolas B, Guo L, Zhou R, Nath K, Nelson DS, Leeper DB (2016). The anti-tumour agent lonidamine is a potent inhibitor of the mitochondrial pyruvate carrier and plasma membrane monocarboxylate transporters. Biochem J.

[40] Ravagnan L, Marzo I, Costantini P, Susin SA, Zamzami N, Petit PX (1999). Lonidamine triggers apoptosis via a direct, Bcl-2-inhibited effect on the mitochondrial permeability transition pore. Oncogene.

[41] Nath K, Guo L, Nancolas B, Nelson DS, Shestov AA, Lee SC (1866). Mechanism of antineoplastic activity of lonidamine. Biochim Biophys Acta.

[42] Orlandi L, Zaffaroni N, Bearzatto A, Villa R, De MA, Silvestrini R (1998). Lonidamine as a modulator of taxol activity in human ovarian cancer cells: effects on cell cycle and induction of apoptosis. Int J Cancer.

[43] Sánchez Y, Simón GP, Calviño E, De BE, Aller P (2010). Curcumin stimulates reactive oxygen species production and potentiates apoptosis induction by the antitumor drugs arsenictrioxide and lonidamine in human myeloid leukemia cell lines. J Pharmacol Exp Ther.

[44] Salliot C, Finckh A, Katchamart W, Lu Y, Sun Y, Bombardier C (2011). Indirect comparisons of the efficacy of biological antirheumatic agents in rheumatoid arthritis in patients with an inadequate response to conventional disease-modifying antirheumatic drugs or to an anti-tumour necrosis factor agent: a meta-analysis. Ann Rheum Dis.

[45] Seki N, Sudo Y, Yoshioka T, Sugihara S, Fujitsu T, Sakuma S (1988). TypeII collagen-induced murine arthritis: induction and perpetuation of arthritis require synergy between humoral and cell-mediated immunity. J Immunol.

