# Supplementary material for: Inhibition of hexokinases holds potential as treatment strategy for rheumatoid arthritis
Source: Arthritis Res Ther. 2019 Apr 3;21:87. doi: 10.1186/s13075-019-1865-3 (PMC6446273; doi:10.1186/s13075-019-1865-3)
Supplement: Supplementary file 1 — Table S1. Primers used in this study. (DOC 31 kb) [file 13075_2019_1865_MOESM1_ESM.doc]

| **Supplementary Table 1 Primers used in this study** | | |
| --- | --- | --- |
| **Gene** | **Forward** | **Reverse** |
| HK-I | CTCTGGGCCTTGAACATT | CCCTCAGTGACTCCAAA |
| HK-II | GTGCTGTACAGGAGCCGAA | TTTCCTTGCTTCCTCCGAGTC |
| HK-III | CAAAGTTGAATCAGAAGATACAAG | CTTCCTCAAGTTGCTGGTC |
| HK-IV | CGAGAAGCATTCCCAACCCT | GGCTCATATCGTAGAGCGGG |
| CXCL9 | AGGTGGCCAAACACTGAGAC | GTGAAGGCCTCTAGTCGCTG |
| CXCL10 | CACAGCTGCCCATTGCCTAAAGAA | GGCCTGTGTCTTCAGGCTCAAA |
| CXCL11 | ATGACGGAACTAGAGACAGCC | AGGAAGCCTGGTAGCTCCTT |
| GAPDH | AATGGGCAGCCGTTAGGAAA | GCGCCCAATACGACCAAATC |
