# Supplementary figures and images for: Inhibition of hexokinases holds potential as treatment strategy for rheumatoid arthritis
Source: Arthritis Res Ther. 2019 Apr 3;21:87. doi: 10.1186/s13075-019-1865-3 (PMC6446273; doi:10.1186/s13075-019-1865-3)

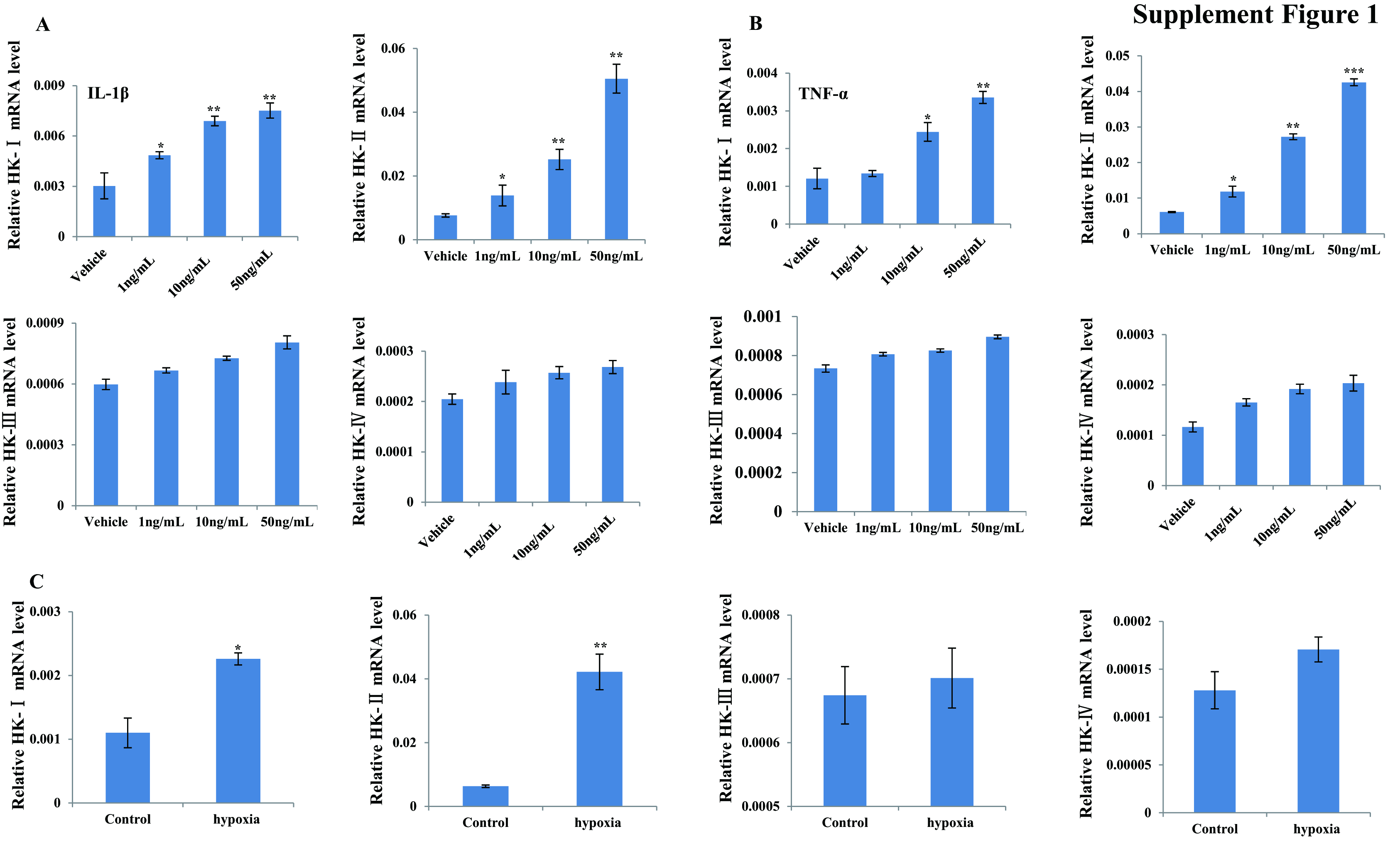

Supplement: Supplementary file 2 — Figure S1. Effects of proinflammatory factors and hypoxia on the mRNA level of HKs. RASFs (n = 3) were treated with IL-1β (A), TNF-α (B) and CoCl2 (C). Then, RT-qPCR was performed to detect the mRNA expression of HK-I, HK-II, HK-III and HK-IV. Results are shown as the mean ± SEM. The statistical significances of differences vs vehicle or control group were determined. *p < 0.05, **p < 0.01 significantly different. (TIF 27758 kb) [file 13075_2019_1865_MOESM2_ESM.tif]

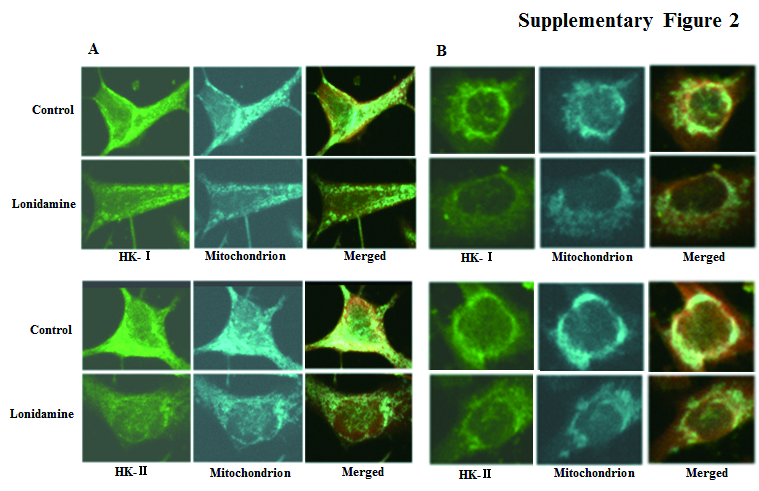

Supplement: Supplementary file 3 — Figure S2. Effects of LND on cellular distribution of HK-I and HK-II. RASFs (A) and THP-1 (B) cells were treated with LND (100 μM) for 24 h. Immunofluorescent staining (green) of HK-I and HK-II in mitochondria (blue) was analyzed. (TIF 2967 kb) [file 13075_2019_1865_MOESM3_ESM.tif]

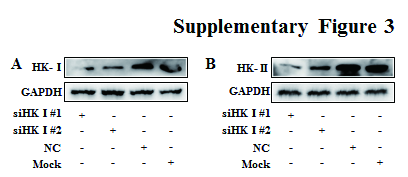

Supplement: Supplementary file 4 — Figure S3. Silencing efficiency of siRNA targeting HK-I (A) and HK-II (B) in THP-1 cells. (TIF 849 kb) [file 13075_2019_1865_MOESM4_ESM.tif]
